# A long‐standing case of desmoplastic fibroblastoma of the face

**DOI:** 10.1002/ccr3.6029

**Published:** 2022-07-11

**Authors:** Arpita Singh, Snehashish Ghosh, Satyapriya Shivakotee, Safal Dhungel, A. Thirumal Raj, Shankargouda Patil

**Affiliations:** ^1^ Department of Oral and Maxillofacial Surgery National Medical College and Teaching Hospital Birgunj Nepal; ^2^ Department of Oral Pathology College of Medical Sciences Bharatpur Nepal; ^3^ Department of Oral and Maxillofacial Surgery Kantipur Dental College and Hospital Kathmandu Nepal; ^4^ Department of Oral and Maxillofacial Surgery College of Medical Sciences Bharatpur Nepal; ^5^ Department of Oral Pathology and Microbiology Sri Venkateshwara Denta College and Hospital Chennai India; ^6^ Department of Maxillofacial Surgery and Diagnostic Sciences, Division of Oral Pathology College of Dentistry, Jazan University Jazan Saudi Arabia

**Keywords:** benign, desmoplastic, female, fibroblastoma

## Abstract

The present case report depicts an unusually large desmoplastic fibroblastoma. The diagnosis of the lesion appears to be deceptive clinically. The purpose of this case image is to highlight its size and presenting symptoms, which could easily be mistaken for an odontogenic, salivary gland, or a soft tissue neoplasm.

## Graphical abstract

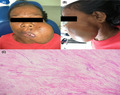

## 1 INTRODUCTION

Evans et al.^1^ in 1995 described desmoplastic fibroblastoma (DF) as a benign soft tissue tumor.^1^ Based on the histopathological observation of Neilsen et al.,^2^ the term collagenous fibroma was suggested for DF.^2^

## 2 CASE PRESENTATION

**FIGURE 1:**
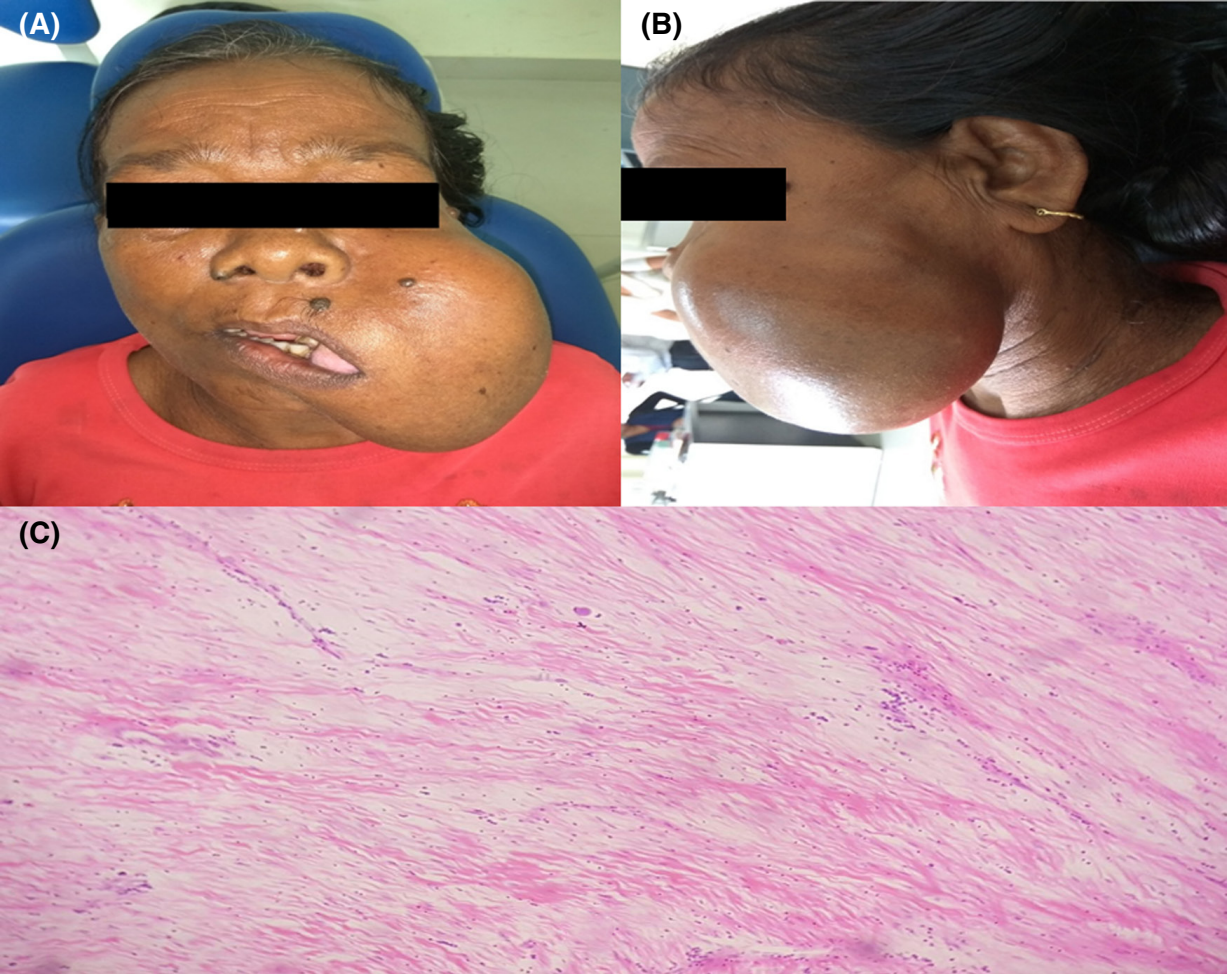
(A) Preoperative frontal view of the patient. (B) Preoperative lateral view of the patient. (C) Photomicrograph showing hypocellular areas with fibroblastic and myofibroblastic proliferation (200× magnification)

A 50‐year‐old woman reported to the outpatient department of Oral and Maxillofacial Surgery with a chief complaint of facial swelling for around 13 years. Following examination, a soft tissue swelling measuring (10x9x2) cm was noted on the left side of the face (Figures 1A,B). The swelling was firm and nontender, and the overlying skin was normal.

## 3 DIAGNOSIS

Microscopic examination from the most dependent part of the lesion revealed a nonencapsulated lesional tissue consisting of proliferation of spindle‐ and stellate‐shaped fibroblasts with large oval nuclei and bi‐ or trinucleation, immersed in the abundant dense collagenous stroma (Figures 1C).

## 4 MANAGEMENT

**FIGURE 2:**
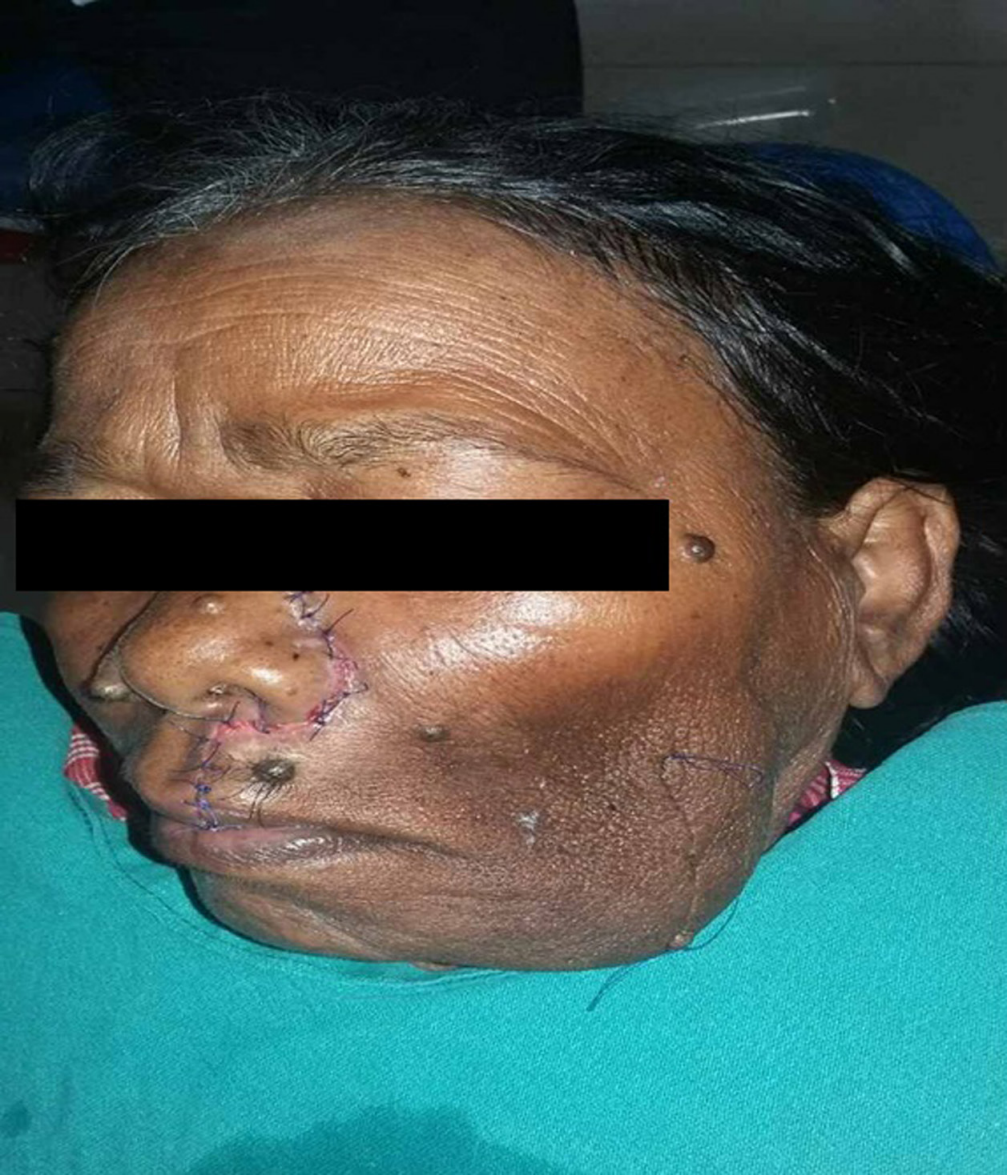
Postoperative view of the patient

Treatment included a wide local excision of the lesion and primary closure under general anesthesia. Following treatment, the patient was observed for 48 hours and then discharged. Postoperative view of the patient is depicted in Figure 2. The patient did not report back for follow‐up. Take‐home message is that whenever such a case is encountered, this entity should be considered one of the differential diagnoses. Histopathology is mandatory before the formulation of a treatment plan to ensure the correct treatment is rendered to the patient.

## AUTHOR CONTRIBUTIONS

All the authors contributed to the writing of the manuscript.

## CONFLICT OF INTEREST

None.

## ETHICAL APPROVAL

Ethical approval was not required from the institution, in accordance with our country's law, as this was a case report.

## CONSENT

Written informed consent was obtained from the patient to publish this case image in accordance with the journal's patient consent policy.

## Data Availability

The data that support the findings of this article are available from the corresponding author upon reasonable request.
